# A role for small secreted proteins (SSPs) in a saprophytic fungal lifestyle: Ligninolytic enzyme regulation in *Pleurotus ostreatus*

**DOI:** 10.1038/s41598-017-15112-2

**Published:** 2017-11-06

**Authors:** Daria Feldman, David J. Kowbel, N. Louise Glass, Oded Yarden, Yitzhak Hadar

**Affiliations:** 1 The R.H. Smith Faculty Agriculture, Food and Environment, The Hebrew University of Jerusalem, Department of Plant Pathology and Microbiology, Rehovot, 76100 Israel; 2 University of California at Berkeley UC Berkeley, Department of Plant and Microbial Biology, 111 Koshland Hall, Berkeley, California, 94720 USA

## Abstract

Small secreted proteins (SSPs), along with lignocellulose degrading enzymes, are integral components of the secretome of *Pleurotus ostreatus*, a white rot fungus. In this study, we identified 3 genes (*ssp1*, *2* and *3*) encoding proteins that are annotated as SSPs and that exhibited of ~4,500- fold expression, 24 hr following exposure to the toxic compound 5-hydroxymethylfurfural (HMF). Homologues to genes encoding these SSPs are present in the genomes of other basidiomycete fungi, however the role of SSPs is not yet understood. SSPs, aryl-alcohol oxidases (AAO) and the intracellular aryl-alcohol dehydrogenases (AAD) were also produced after exposure to other aryl-alcohols, known substrates and inducers of AAOs, and during idiophase (after the onset of secondary metabolism). A knockdown strain of *ssp1* exhibited reduced production of AAO-and AAD-encoding genes after HMF exposure. Conversely, a strain overexpressing *ssp1* exhibited elevated expression of genes encoding AAOs and ADD, resulting in a 3-fold increase in enzymatic activity of AAOs, as well as increased expression and protein abundance of versatile peroxidase 1, which directly degrades lignin. We propose that in addition to symbionts and pathogens, SSPs also have roles in saprophytes and function in *P. ostreatus* as components of the ligninolytic system.

## Introduction

Fungi secrete proteins that participate in polymeric organic matter degradation, such as proteases, lipases and carbohydrate-active enzymes^1^ or facilitate interactions through surface proteins such as hydrophobins, or small-secreted proteins (SSPs)^2,3^. In fungi, SSPs are characteristically shorter than 300 amino acids and have a secretion signal peptide^3^. SSPs are mostly present in fungi that interact with living plants and some of these SSPs have been referred to as ‘effectors’, which are key factors in plant infection that are involved in modulating plant response to infection^1^. In saprophytic fungi, SSPa have been suggested to be involved in degradative capabilities, as in the case of the *Trichoderma reesei* swollenin protein, which mediates depolymerization of cellulose^4^. SSPs are mostly encoded by orphan genes with no known PFAM (protein families database) domains. Only a few SSPs have been functionally characterized^2,3^.

In a previous study, we explored the ability of *Pleurotus ostreatus*, a white-rot fungus, an efficient degrader of lignocellulose and a variety of aromatic compounds, to metabolize 5-hydroxymethylfurfural (HMF)^5^. This toxic compound is generated during the pretreatment of cellulosic biomass for 2^nd^ generation biofuel production, and is considered as a key technical challenge in the process^6–9^. We demonstrated that *P. ostreatus* bioconverts HMF via enzymes involved in lignin degradation, including the extracellular H_2_O_2_-generating aryl-alcohol oxidases (AAO) and the intracellular aryl-alcohol dehydrogenases (AAD)^10,11^. Other important lignin-modifying enzymes are the extracellular metal-containing oxidoreductases, which include enzymes that utilize hydrogen peroxide, manganese peroxidases [MnPs, as well as versatile peroxidases (VPs)]^12–14^, lignin peroxidase, laccase and dye peroxidase^10^.

In the current study, we identified genes encoding a family of *P. ostreatus* SSPs, in whose expression was upregulated in response to exposure to HMF or other aryl alcohols. The expression of these SSP coding genes was also increased, even in the absence of the inducer, at the onset of idiophase (the growth phase in which secondary metabolism occurs). Genetic manipulation of SSP-coding genes, by introduction of either knockdown or overexpression cassettes into *P. ostreatus* resulted in effects on AAOs, AAD and VP, at the transcriptional, translational and enzyme activity levels. We suggest that the SSPs function to facilitate induction of the ligninolytic system, although the nature of the regulatory pathway associated with induction is yet unclear. Most analyses of SSP function have focused on their involvement in pathogenic or symbiotic lifestyles of various fungi. We now demonstrate that SSPs have a function during the onset of activity of the ligninolytic system in a fungus whose lifestyle is completely saprophytic.

## Results

### Small Secreted Protein (SSP) expression is induced after exposure to HMF

Previously, we reported on the involvement of the lignin-modifying enzymes AAO and AAD in the transcriptional response to HMF, as well as in its degradation^5^. To determine if other genes associated with the ligninolytic process are potentially affected by the presence of HMF, we performed RNA-seq analysis on 5-day-old cultures exposed to HMF for 24 hrs. The expression of 15 out of 39 genes encoding AAOs, and 6 genes out of 8 encoding AADs was significantly increased. Among the 9 members of the MnP family, which also consists of VP enzymes^12–14^, only the expression of *mnp1* was significantly increased (~2-fold; Supplementary Table S1). In addition, expression of two laccase encoding genes was affected: PoLACC6 (POXA1B) and PoLACC3, which were both upregulated (~3.8 and 2.2 fold, respectively) (Table S1). A summary of all genes whose expression was affected by the addition of HMF can be found in Table S2.

In addition to the expected and previously described changes in gene expression^5^, a marked increase in the expression of 3 genes that encode for small secreted proteins was evident. The 3 genes: 65712, 46202 and 91630 were upregulated between 250-900-fold, relative to the control. The genes share a high degree of similarity and are predicted to encode small (~18 kDa) proteins. Bioinformatic analyses revealed that the identifiable conserved motif (present in the three proteins) was a signal peptide. Similar proteins, found in other fungi, have been designated SSPs (Small Secreted Proteins)^2^. Thus, we designated the mentioned *P. ostreatus* proteins *ssp1*, *2* and *3*, respectively. The expression level of three additional genes in the *P. ostreatus* genome that belong to the same family (90499, 44261 and 100993, designated *ssp4*, *5*, and *6*) was not affected.

Homologues of the *P*. *ostreatus* SSP family are present in the genomes of other fungi (Table S3)^15,16^, mostly in basidiomycete species in the Agaricomycotina, such as *Pleurotus eryngii* and *Coprinopsis cinerea*, where their function is also unknown. Ssp1 is highly similar (E value of 4.39E-023) to the *Laccaria bicolor* MISSP24 (protein id 293250). MISSP24 was annotated as a small putative secreted protein, which is up-regulated during ectomycorrhizal growth of *L. bicolor* in Populus and Douglas fir and in fruiting bodies of the fungus (http://genome.jgi-psf.org/Lacbi2/Lacbi2.home.html). Though *P. ostreatus* is not an ectomycorrhizal fungus, it may suggest that SSPs play a role in *P. ostreatus’* natural habitat as well and suggest a conserved function with other SSPs-containing fungi^1,2^.

**Figure 1:**
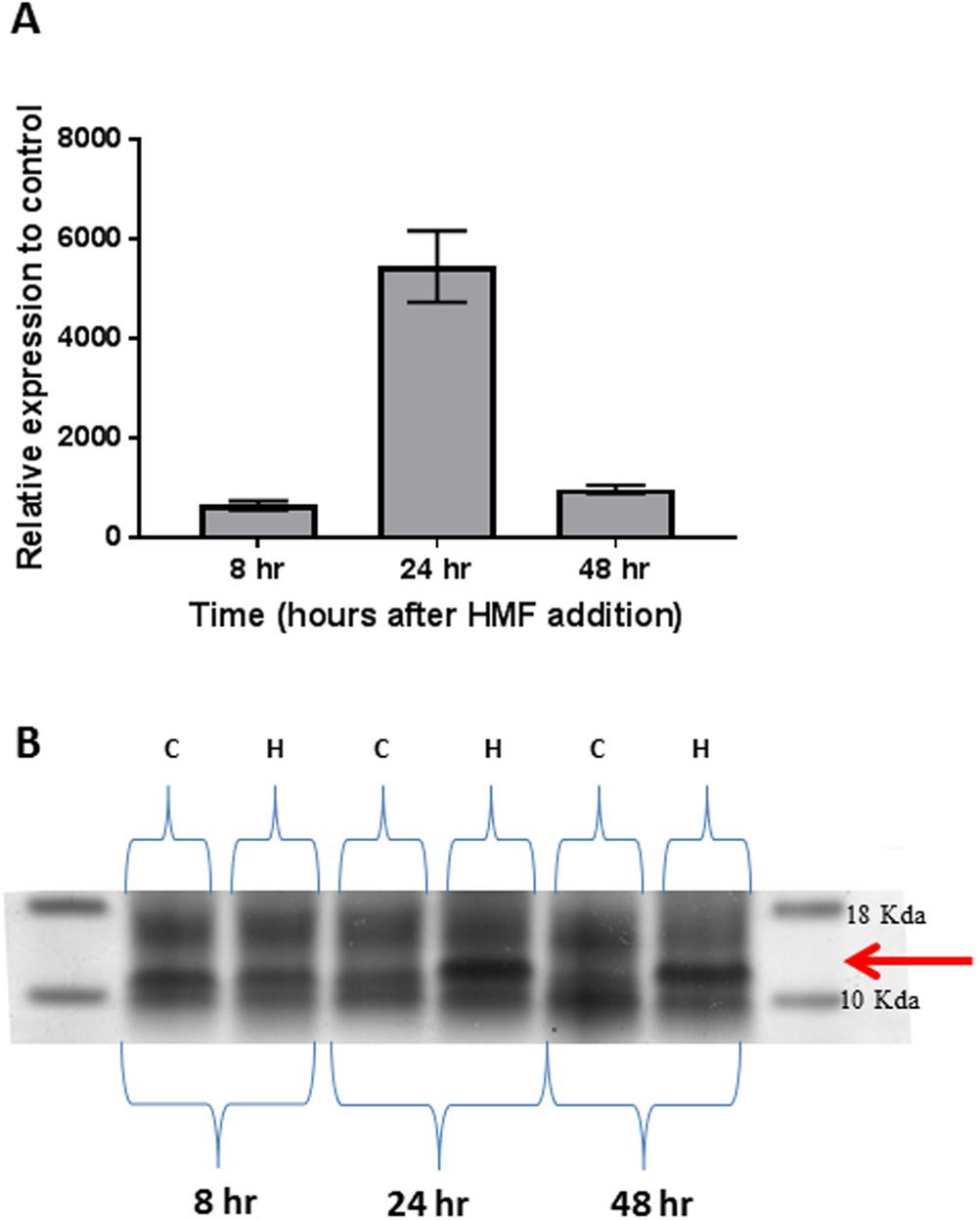
Elevated gene expression and protein abundance of Ssp1-3 in *P. ostreatus* following HMF exposure. The expression of *ssp*s relative to control (**A**) and secreted protein abundance of SSPs,relative to total secreted proteins (**B**), were analyzed 8, 24 and 48 h after addition of 30 mM of HMF to the media. The identification is based on MS. Arrow points to SSPs.

To verify the results obtained by the RNA Seq procedure and further monitor the induction of SSP over time after exposure to HMF, we constructed specific primers for RT- PCR. The primers were constructed on the basis of the *ssp1* gene, but due to the high similarity of *ssp1* to the other *ssp* genes in the family we regarded the amplicon obtained, which contained a mixture of at least three gene products, as the collective expression of *ssp1*, *2* and *3*. When the cultures were not exposed to HMF, the expression of all *ssp*s was very low, 5*10^−04^ relative to β-tubulin in 5–7-day-old cultures. A 4-fold increase in the expression of *ssp* genes occurred within 30 minutes after addition of 30 mM HMF, followed by a ~650-fold and ~5550-fold increase after 8 and 24 hours, respectively. After 48 hours of exposure to the furan, the expression of the *ssp* genes was reduced to ~950-fold, relative to the control (0.11 relative to β-tubulin) (Fig. 1). The time points corresponded to the time course of biotransformation of HMF by *P. ostreatus*: the extracellular concentration was reduced by ~10% after 8 hours, 50% reduction by 24 hours, and complete biotransformation occurred after 48 hours^5^. These results suggested that the level of the induction of *ssp* genes correlated with the amount of HMF in the medium, as well as with its degradation kinetics by the fungus.

In order to verify that the increase in RNA levels leads to accumulation of the predicted SSP proteins, the fungal secretomes were resolved by SDS-PAGE. We observed a high level of ~15 kDa proteins (corresponding to the expected size of the *ssp* gene products), which were present only in medium from cultures that had been exposed to HMF for 24 or 48 hrs (Fig. 1). To determine the identity of the SSPs in the secretome of *P. ostreatus*, we analyzed the relevant PAGE bands by mass spectrometry (MS) and identified the corresponding proteins, Ssp1 and Ssp2, at a very high abundance. Though the expression of *ssp* genes was highly induced 8 hr after exposure to HMF (Fig. 1), we only detected the presence of the corresponding proteins at later time points. A possible explanation for this may be the time required between the transcription, translation/secretion and accumulation of these SSP proteins in the media.

### SSPs, AAOs and AAD are induced by exposure to aryl-alcohols

**Figure 2:**
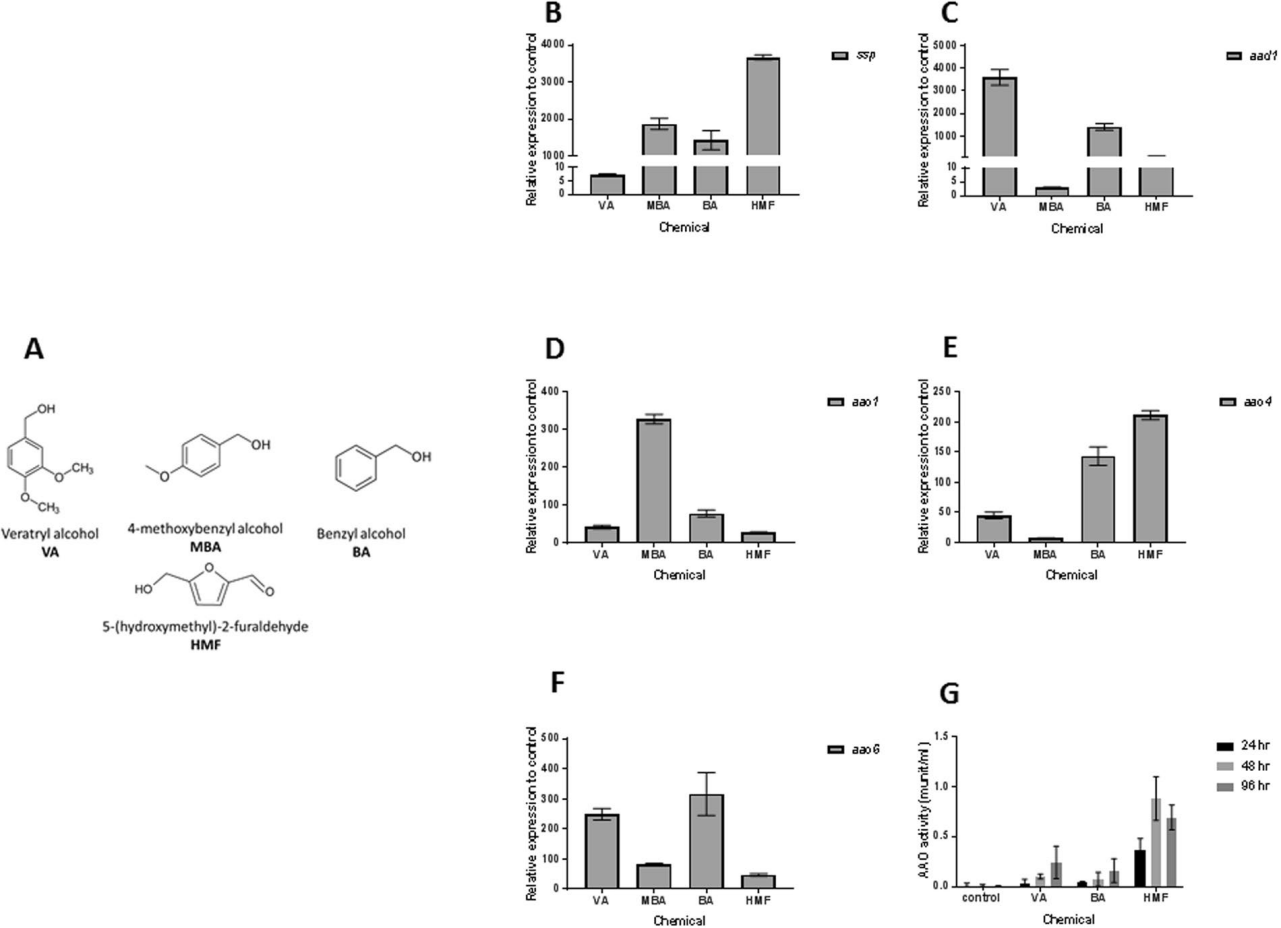
Elevated gene expression of *ssp*s, *aao*s and *aad* and increased *in vitro* AAO activity following exposure to aryl-alcohols and HMF. (**A**) Chemical structure of Veratryl alcohol (VA), 4-methoxybenzyl alcohol (MBA), benzyl alcohol (BA) and HMF (**B**) 30 mM of VA, 4- MBA, BA or HMF were added to *P. ostreatus* media. Relative gene expression to control of *ssp*s (**B**), *aad1* (**C**) and *aao1,4*, and *6* (**D–F**) was analyzed following 24 hours exposure to the chemicals. *In vitro* AAO activity in free cell extracts (**G**) was measured 24, 48 and 96 hours following the chemicals addition.

The AAO and AAD gene family members are involved in HMF biotransformation, and involved in the transcriptional and translational response to the chemical in *P. ostreatus*^5^. As the expression of these two gene families in response to HMF coincides with that of the SSPs, we hypothesized that the induction and accumulation of SSPs might be linked with HMF metabolism. To explore this possibility, we chose several known substrates or inducers of AAOs, and determined the transcriptional and translational response of AAOs following the addition of 30 mM of each chemical. We analyzed the fungal response to 3 aryl alcohols: veratryl alcohol (VA), 4-methoxybenzyl alcohol (MBA) and benzyl alcohol (BA) (Fig. 2). All the chemicals induced *ssp* gene expression, as measured 24 hr after their addition to the media. The most significant induction occurred after the addition of HMF (~3670-fold), followed by BA and MBA (~1425 and ~1865 fold, respectively), and only ~7-fold in VA-amended medium (Fig. 2). The transcriptional induction of the *ssp1-3* was coupled with induction of genes encoding AAO1-3 and AAD1, by exposure to all chemicals, albeit at different levels (Fig. 2). AAO activity, as measured calorimetrically, was induced by the presence of VA and BA, but 4-fold less than by HMF (after the addition of MBA, AAO activity could not be measured due to technical limitation of the assay) (Fig. 2). Additional compounds, tested as controls, such as other furans (furfural and furfuryl alcohol) and a known inducer of laccase (guaiacol) did not trigger the same reaction in gene expression.

Elevated expression of genes encoding SSPs was coupled with elevated expression of genes encoding AAOs and AAD when exposed to HMF, VA, BA or MBA. These chemicals are toxic to *P. ostreatus* at the concentrations used, and significantly reduced the growth rate of the fungus. All the molecules tested share a common aryl alcohol moiety, or in the case of HMF (Fig. 2), a C-OH bond to a ring, which we suggest is probably mandatory, but not the only functional group, required for triggering the response.

### SSPs gene expression is induced during the idiophase

**Figure 3:**
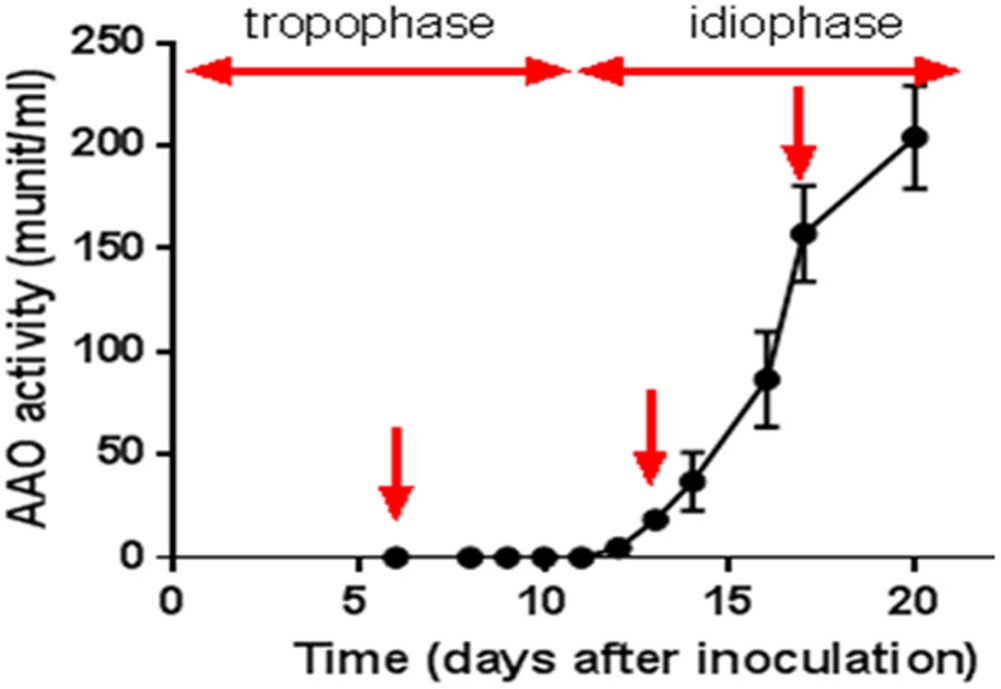
*In vitro* AAO activity at different growth stages of *P. ostreatus*. The activity of AAO was monitored in free cell extracts and was measured over 21 days. Arrows point to 6, 13 and 18-day-old cultures that were further analyzed.

The similar reaction of the *ssp*s, *aao*s and *aad* genes to the same molecules, which can also all serve as a substrate for AAO, led us to hypothesize that the 3 gene families might be expressed under the same conditions. We measured AAO activity at different growth stages for over 24 days of growth, to be used as a potential marker to identify the culture conditions under which these genes are expressed. During the fungal growth phase (the first 10–11 days following inoculation), AAO activity was not detected. 12–13 days following inoculation, at the beginning of the idiophase, as the fungus enters a stage where secondary metabolism occurs^17^, there was a significant increase in AAO activity. We focused on 6, 13 and 18-day-old cultures, representing cultures lacking detectable AAO activity, an early AAO induction phase and a high activity phase, respectively (Fig. 3).

**Figure 4:**
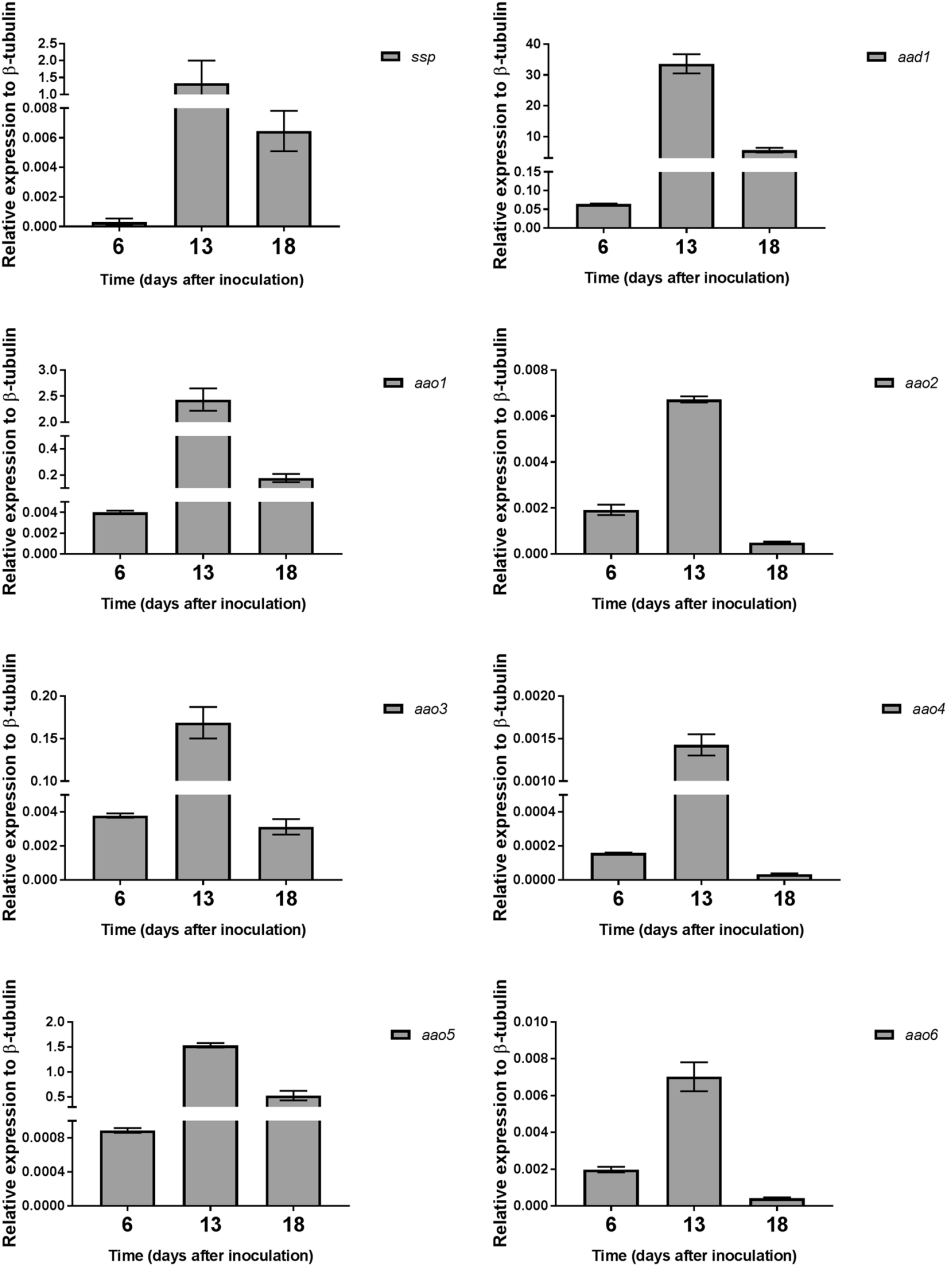
Gene expression of *ssp*s, *aao*s and *aad* at different developmental stages. Expression levels relative to β-tubulin gene of *ssp*s, *aad1* and *aao1-6*, measured at 6 (early growth stage), 13 (idiophase) and 18 (late idiophase) day-old cultures of *P. ostreatus*.

**Figure 5:**
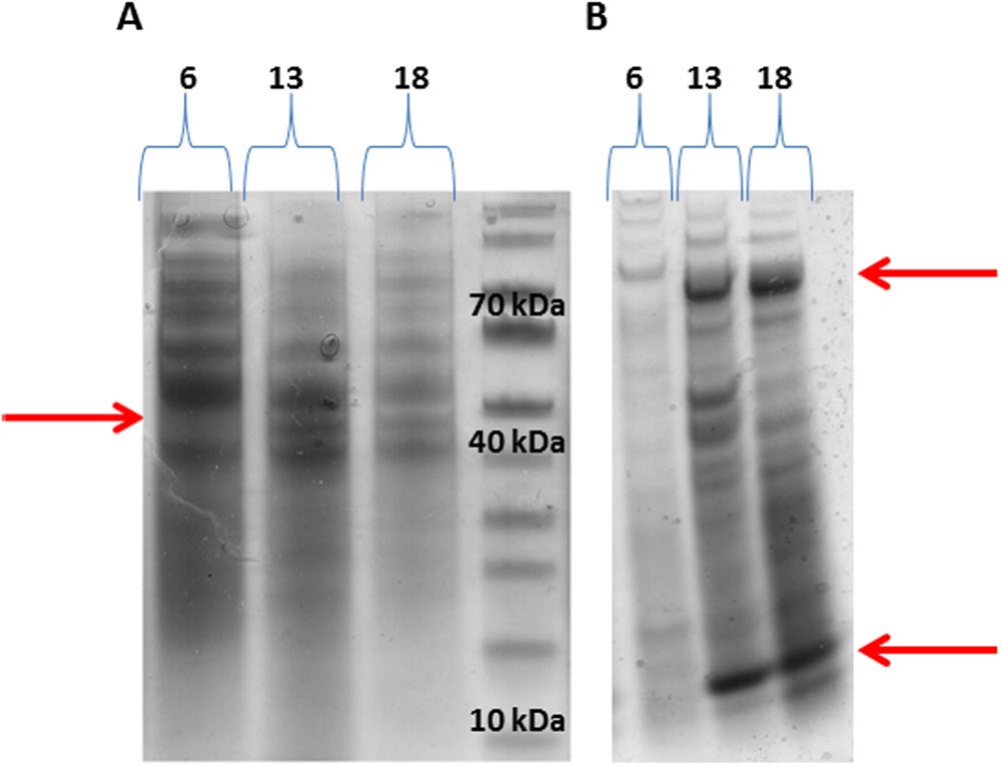
Profiles of secreted and cellular *P. ostreatus* proteins at different developmental stages. Cellular (**A**) and secreted (**B**) proteins were extracted from *P. ostreatus* at 6 (early growth stage), 13 (idiophase) and 18 (late idiophase) day-old cultures. Arrows point to AAD (**A**), AAO (B-up) and SSP (B-down). Protein identification was confirmed by MS analysis.

At day 13, the culture showed the highest level of *ssp* transcript abundance (1.33 relative to β-tubulin), which decreased after 18 days (0.0057 relative to β-tubulin) (Fig. 4). At the protein level, high amounts of SSPs were detected on both days 13 and 18, and were accompanied by high levels of AAOs and AAD protein abundance in the secreted and cellular fractions, respectively (Fig. 5).

### Knockdown of SSP results in reduced expression of AAO and AAD after HMF exposure

In an attempt to reveal the function of SSPs in *P. ostreatus*, we took a reverse genetics approach to determine if silencing the corresponding genes would result in altered sensitivity to HMF. Specifically, we generated an RNAi construct designed to interfere with the expression of the entire *ssp* gene family. The plasmid was constructed by incorporating a highly conserved 160-bp *ssp* gene fragment (based on the sequence of *ssp1*) arranged in a forward and a reverse orientation, where the *ssp1* fragment flanks a 213 bp loop. This construct was inserted into pTMS1 [which harbors a carboxin resistance cassette^13^], downstream of the constitutive *Sdi1* promoter^13,18^. The resulting plasmid (pDF3) was used to transform *P. ostreatus*. Carboxin-resistant transformants were further analyzed, by PCR, to verify the presence of the insert.

**Figure 6:**
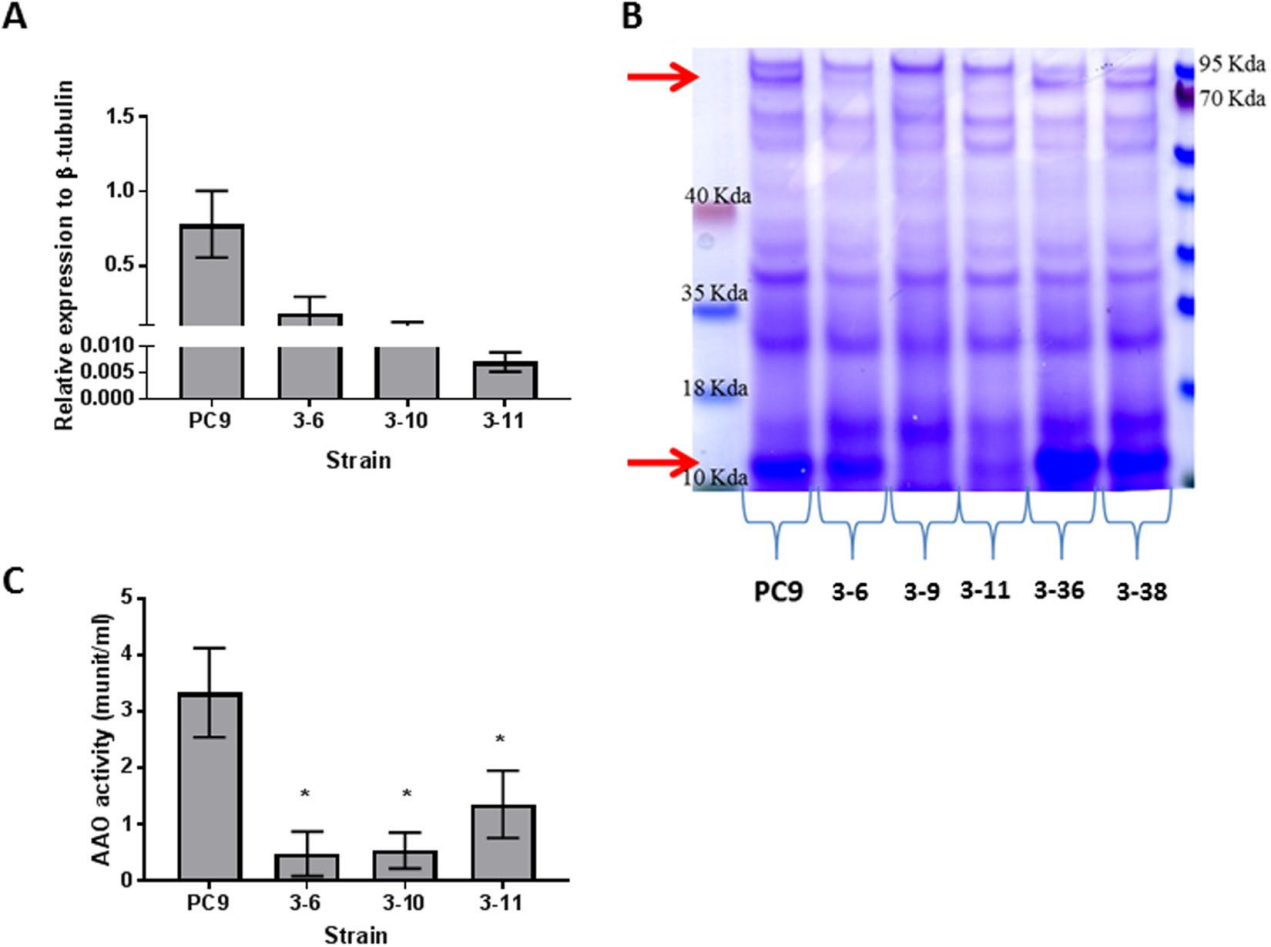
Reduced expression of *ssp*s and AAO activity in a KD*ssp1* background, following HMF exposure. The expression and AAO activity of *ssp1* knockdown transformants (3–8, 3–10 and 3–11) relative to the parental strain, PC9 was analyzed 24 hours after addition of 30 mM of HMF. (**A**) Expression level of *ssp*s relative to β-tubulin. (**B**) Profiles of secreted proteins. Arrows point to AAO (up) and SSP (down). (**C**) *In vitro* AAO activity in free cell extracts.

We analyzed the SSP-induction response of 5 transformants, 24 hours after exposure to HMF. The secreted proteins were resolved on an SDS-PAGE gradient gel, stained with Coomassie Blue, and the amount of SSPs examined. A clear reduction in SSPs was observed in transformants 3–6, 3–10 and 3–11, while transformant 3–36 and 3–38 exhibited SSP levels which were similar to the PC9 control strain (Fig. 6B). The differential expression of *ssp*s was typical to RNAi transformants^13,19^. Based on these results we chose to focus our further analyses on transformants 3–6, 3–10 and 3–11.

We quantified the expression of *ssp*s 24 hr after exposure to HMF. The expression level was significantly decreased, by 4–110 fold, in the transformants, as compared to the PC9 control strain (Fig. 6A). When the wild type fungus is grown in the absence of HMF the expression of *ssp*s is negligible, the comparative decrease in expression levels of the transformants was difficult to quantitate.

The AAO accumulation was significantly reduced and barely detected in the RNAi transformants, relative to the PC9 strain (Fig. 6B). Furthermore, the levels of AAO activity seem to correlate with SSPs levels (Fig. 6C). The AAO activity level in the PC9 strain after exposure to HMF was ~3.5 munit/ml, while the AAO activity of all the 3 transformants analyzed was significantly reduced by an average of ~5-fold, to ~0.5–1.3 munit/ml (Fig. 6C). To examine whether the reduction in AAO activity was a result of changes in transcription of the relevant genes, we quantified the expression of *aao1-6*, encoding for the six AAOs in the *P. ostreatus* genome. The expression of *aao4* and *aao5* was reduced ~2-20-fold in all the 3 transformants. Transformants 3–6 and 3–11 also exhibited a ~4-fold reduction in *aao1* and *3* expressions. The expression of the AAD coding gene *aad1* was also reduced ~2-fold in 3–6 and 3–10, and ~5.5-fold in 3–11. We decided to further characterize transformant 3–11, referred to as KD*ssp1*, which did not exhibit phenotypic alterations except reduced growth rate of about 10% relative to parental strain.

Based on the results it seems that knockdown of *ssp* showed reduced expression of AAOs and AAD, after exposure to HMF.

### Overexpression of SSP results in elevated expression of AAO and AAD

**Figure 7:**
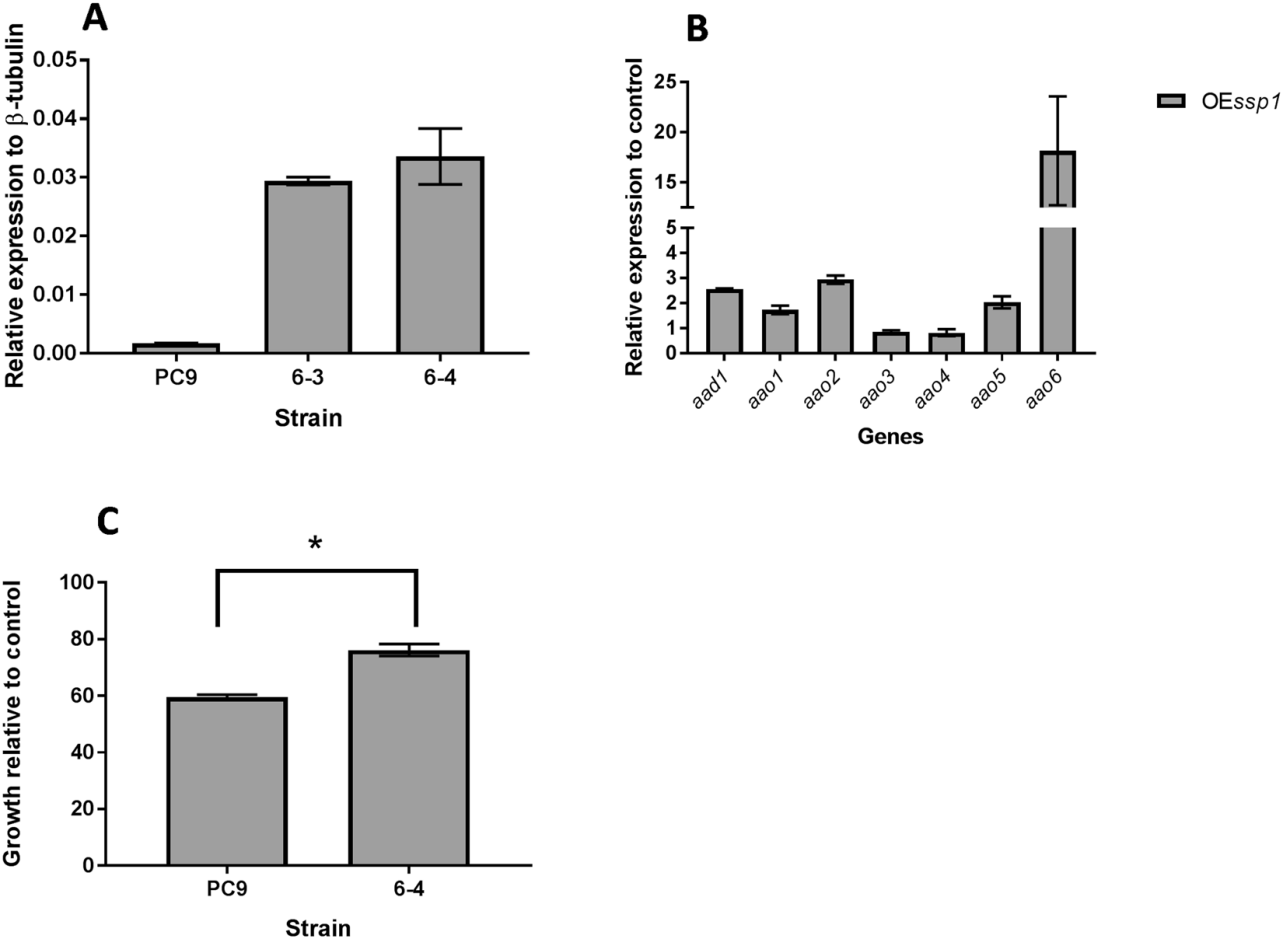
The over-expression of *ssp1* results in elevated expression of *aao1*-*6* and *aad1* increased tolerance to HMF. The expression level of *ssp1* in transformants bearing an *ssp1* over-expression construct (**A,B**) was analyzed in a 7-day-old culture. (**A**) *ssp*s expression relative to β-tubulin. (**B**) Expression levels of *aad1* and *aao1-6* in a 6–4 strain background, relative to the expression levels of these genes in the parental PC9 strain. (**C**) Relative colony growth of 8-day-old cultures of the parental PC9 strain compared to the *ssp1* over-expression strain (6–4) after addition of 15 mM HMF.

To determine what the consequences of over-expression of *ssp*s, we produced a *P. ostreatus* strain the overexpressed *ssp1*. A plasmid containing the *ssp1* gene regulated by the β-tubulin promotor and terminator (pDF6) was transformed into *P. ostreatus* PC9, and the presence of the insert in resulting 4 transformants was verified by PCR. Preliminarily analysis was performed on transformants 6–3, 6–4 and 6–10 which exhibited significant elevation in *ssp*s expression. Transformant 6–4 (designated OE*ssp1*) was chosen for further analysis. This strain exhibited ~20-fold elevated expression of *ssp* (Fig. 7), and significantly increased SSP abundance at a 10 days culture, as measured by label free MS (data not shown). The strain exhibited small reduction of ~10% in growth rate relative to PC9.

To examine whether the elevated expression of the *ssp*s affected expression of AAO and AAD encoding genes, we quantified the expression of *aao1-6* and *aad1* in the OE*ssp1* strain in 7-day-old cultures, a time point corresponding to the fungal growth stage. The expression levels of *aao2, aao5*, *aao6* and *aad1* were elevated (~3, ~2, ~18 and 2.5-fold, respectively). However, the expression level of *aao2* was not altered (Fig. 7). We concluded that the expression of at least part of the ligninolytic machinery was, indeed, affected by changes in SSP1 abundance. That and more, the OE*ssp1* was more tolerant toward HMF by ~20% (Fig. 7).

**Figure 8:**
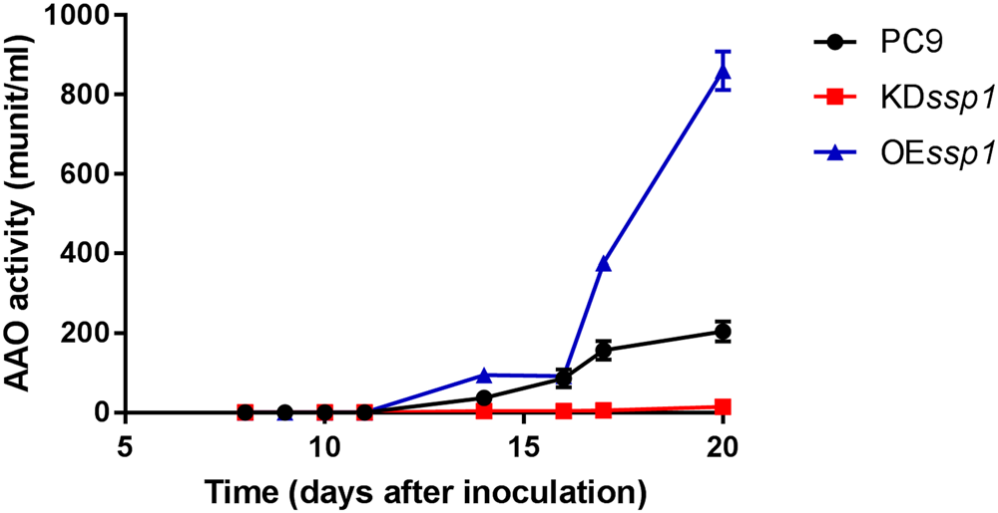
*In vitro* AAO activity is influenced in OE*ssp1* and KD*ssp1* strains. The extracellular activity of AAO was monitored in cell free extracts for 21 days in PC9, OE*ssp1* and KD*ssp1* strains.

To analyze the physiological effects of the KD*ssp1* and OE*ssp1* strains, we monitored AAO activity along the different stages of fungal growth for 28 days. The OE*ssp1* strain exhibited measurable AAO activity (0.2 munit/ml) as early as 8 days in culture, 4 days before the PC9 parental strain (0.033 munit/ml). The AAO activity measured in the OE*ssp1* strain was elevated by ~3–5-fold relative to the PC9 strain during the idiophase, reaching ~700 munit/ml and ~200 munit/ml, respectively in a 20-day-old culture. The opposite effect was observed in the KD*ssp1* strain; where the AAO activity was significantly reduced by ~10–30-fold, and reached only ~14 munit/ml in a 20 day-old-culture (Fig. 8).

**Figure 9:**
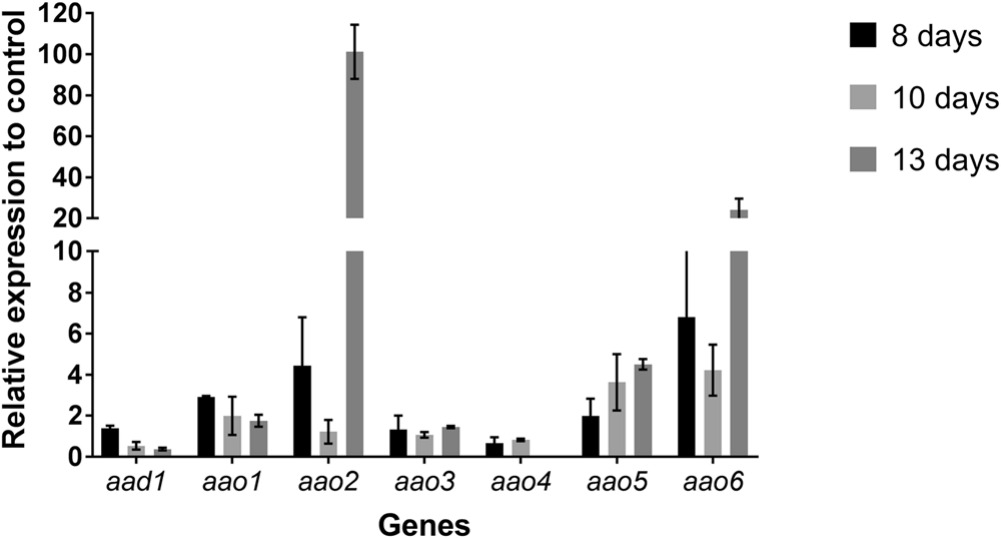
Elevated expression of *aao*s in a KD*ssp1* background at different growth stages. The expression levels of *aad1* and *aao1-6* was determined relative to expression level of these genes in the PC9 and KD*ssp1* strains from 8, 10 and 13-day-old cultures.

Surprisingly, in the KD*ssp1* strain, the reduced protein abundance and AAO activity levels did not correlate with transcription of the relevant genes [this was also evident in the second KD*ssp1* transformant (3–10)]. The expression levels of *aao1, 2, 5* and *6* were elevated at 8, 10 and 13 days of culture relative to the PC9 strain (Fig. 9), suggesting non linearity between the translational and transcriptional response.

### Changes in SSPs influence the abundance of versatile peroxidase 1 (VP1)

**Figure 10:**
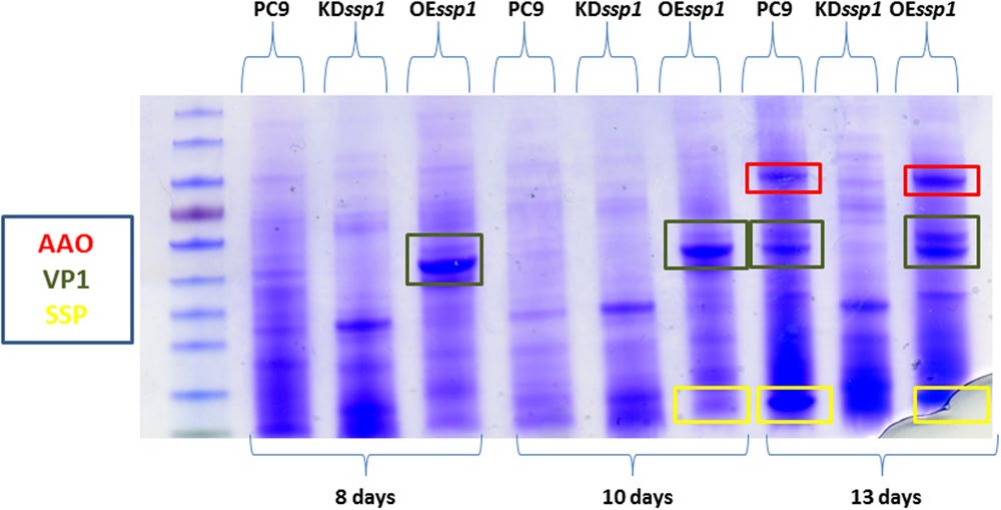
Profiles of secreted proteins at different growth stages in the OE*ssp1* and KD*ssp1* strains. SDS PAGE gel of secreted proteins from 8, 10 and 13-day-old cultures of the PC9, OE*ssp1* and KD*ssp1* strains. Significant differences are marked: AAO (red), VP1 (orange) and SSP (yellow), which were confirmed by MS analysis. The gel is representative to biological triplicates.

To further examine the effect of the KD*ssp1* and OE*ssp1* strains on the secreted proteins over time, we analyzed the relative abundance of secretome at 8, 10 and 13-day-old cultures. In the OE*ssp1* strain, the most striking effect was observed in the abundance of 40–50 KDa proteins, as early as in an 8-day-old culture (Fig. 10). The bands corresponding to this size range were excised from the SDS-PAGE gel and subsequently identified by MS as VP1, (versatile peroxidase 1), a predominant enzyme shown to be involved in lignin modification in Mn^+2^ - deficient media^12,20^. VP1 was identified in the secretome as a visible band in older (13 days) PC9 cultures, yet was almost undetectable in the KD*ssp1* strain (Fig. 10), suggesting the induction of VP1 expression in the OE*ssp1* strain, and the converse when SSP production was decreased.

**Figure 11:**
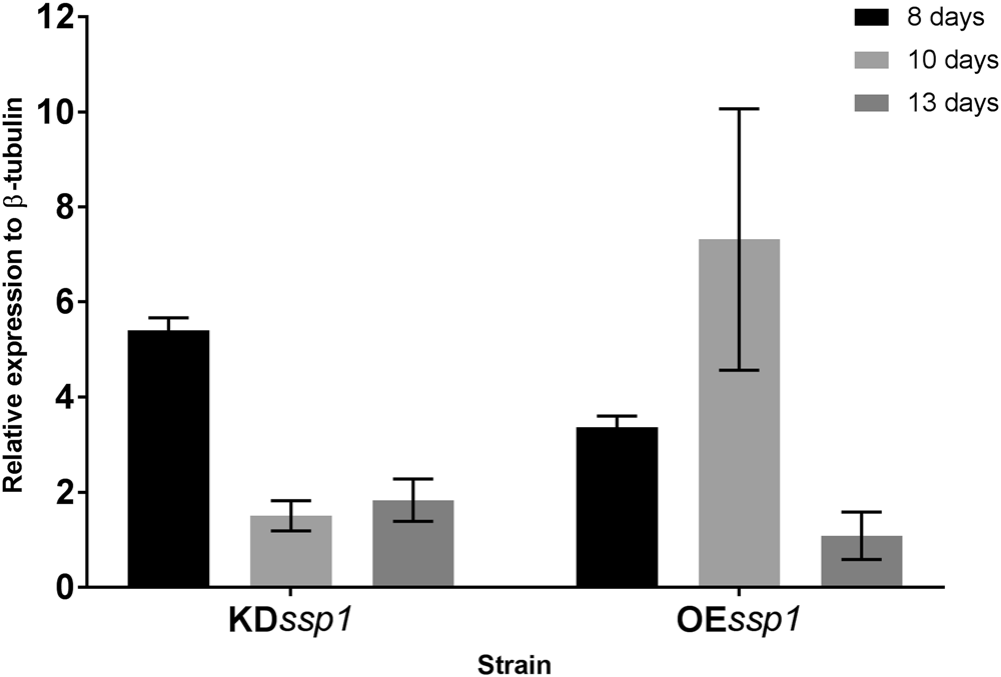
Expression of *vp1* influenced in OE*ssp1* and KD*ssp1* strains. The *vp1* expression levels in the KD*ssp1* and OE*ssp1* strains relative to *vp1* expression levels in the PC9 strain from 8, 10 and 13-day-old cultures.

We monitored the expression level of *vp1* in PC9, KD*ssp1* and OE*ssp1* at the corresponding time points. In the OE*ssp1*, the expression of *vp1* was elevated in 8 and 10-day-old cultures (by ~3.5- and ~7-fold relative to the PC9 strain, respectively), but without significant difference at day 13. Surprisingly, *vp1* transcript levels were also elevated in KD*ssp1* by ~5-fold after 8 days, but not 10 or 13 days, though the VP1 protein was not visible (on the basis of the Coomassie Blue - stained gel) in the secretome of that strain (Fig. 11).

## Discussion

Understanding the functions of SSPs in fungi is still at its early stages, and considered the least characterized component of the secretome^2,3^. Nonetheless, a variety of possible functions for SSPs have been suggested, including the involvement in pathogenicity, symbiosis and degradative capabilities^1,2,21–23^. The production of SSPs is associated with lifestyles and ecological niches; biotrophs and symbionts usually have a higher proportion of species-specific SSPs than hemibiotrophs and necrotrophs^3^. SSPs have also been identified in saprophytic fungi and some of them resemble effector proteins of pathogenic fungi, suggesting alternative roles of SSPs^3^. Here, we present the analysis of a conserved, yet uncharacterized group of SSPs present *in P. ostreatus*, as well as other basidiomycete. Most of the species are from the taxa Agaricomycotina such as *Pleurotus eryngii* and *Coprinopsis cinerea*, in which their function is also unknown. In a majority of the fungi there are multiple copies of the gene (2–12 genes) (Table S3), and a motif of secreted protein, similar to *P. ostreatus*. The *P. ostreatus ssp*s were first identified as small secreted proteins, and the genes were upregulated to a level of up to 4500-fold following exposure of the fungus to HMF (Fig. 1). Furthermore, their corresponding protein products were visibly elevated (on the basis of Coomassie Blue stained gels) in the fungal secretome following exposure to the furan (Fig. 1). These findings triggered our curiosity to further understand their potential function in the fungus.

It was suggested in *Aspergillus* sp, that SSPs may be involved in the fungal strategy to cope with toxicity of aromatic compounds or reactive oxygen species^22^. In the ligninolytic fungus, *Phanerochaete chrysosporium*, several genes coding for SSP were induced in the presence of toxic oak extracts^24^. Since the inhibitory compound, HMF is not present in the natural habitat of *P. ostreatus*, we hypothesized that other chemicals trigger a similar reaction. AAOs are responsible for detoxification of HMF, and their activity is induced following exposure to the compound^5^. We therefore focused our efforts on other aryl-alcohols, known as inducers and substrates of AAOs as well as their prevalence as a functional group of the lignin molecule^25^. Indeed, aryl-alcohols strongly induced the expression of *ssp*s, as well as the expression of *aao*s and *aad* (Fig. 2), thus suggesting a functional link between these three gene families. In culture media, in the absence of chemical exposure, SSPs, AAOs and AAD exhibited elevated expression only during the idiophase (Fig. 4). Activity of ligninolytic enzymes, considered as secondary metabolism, was previously shown during idiophase in *P. chrysosporium*^26^. Recently we have demonstrated that the ligninolytic enzyme-encoding mnp gene family as well as *vp*s is also expressed during the same phase in P. ostreatus PC9^12^. Genetic manipulation of *ssp* expression resulted in an effect on AAOs and AAD. The KD*ssp1* strain exhibited reduced expression of *aao*s and *aad*, after exposure to HMF (Fig. 6), and AAO activity during idiophase was almost abolished (Fig. 8). On the other hand, in the OE*ssp1* strain, elevated expression of *aao*s*, aad* and *vp1* was detected (Fig. 7), AAO activity was increased by 3-fold (Fig. 8), and tolerance toward HMF increased by ~20% (Fig. 7). Based on the genetic and biochemical/physiological data presented, we propose that SSPs may function as regulators, at least in part, of the ligninolytic system. This is based on the evidence presented demonstrating the transcriptional, translational, and enzyme activity responses of several key ligninolytic system components (e.g., AAO, AAD and VP) following manipulation of *ssp* expression.

We suggest that aryl-alcohol/HMF induce the production of SSPs, which are involved in the induced production of AAOs, AADs and VP that are responsible for the modification of lignin. In the presence of HMF, KD*ssp1* exhibit significant reduction in expression, as well as protein abundance of AAO and AAD (Fig. 6), suggesting SSPs may influence the transcription of those genes. Without the external addition of chemical inducers, regulation of SSPs is a bit different. The KD*ssp1* strain exhibited an unexpected phenomenon, in which *aao*s and *vp1* expression levels were elevated, yet the protein abundance of the ligninolytic enzymes was significantly reduced, and AAO activity is almost abolished. An explanation for this could be that the fungus transcribes an excess amount of transcripts of the relevant genes, but this abundance does not result in higher protein synthesis. Though surprising for a secreted protein, it may suggest the SSPs function at the protein level (either by preventing transcription or involved in degradation of proteins) and that the differences observed between transcript and protein abundance may be due to other regulating components at the transcriptional level that function as a feed-back to the reduction/lack of the enzymes.

We are intrigued how can SSPs, being secreted proteins without known catalytic or interacting domains, function as regulators of three large enzyme families. We explored the possibility that SSPs are part of a protein complex, which other members are important for regulation of transcription and translation. In *Aspergillus* sp, it was suggested that SSPs could directly interact with the enzymes to increase their activity^22^. Although utilizing different approaches (including co-immunoprecipitation and yeast 2-hybrid libraries for the screening of SSP-interacting proteins) did not yield conclusive results, our lack of success does not refute this hypothesis. The eventual success in finding SSP-interacting proteins may prove to be the key to delineate the pathway by which the observed SSP-based regulation occurs. Another possibility is that SSPs function by interacting with metabolites, and function as a “bridge” for signaling or interaction with other components. Possible metabolites may be the aryl-alcohols, which induce the production of SSPs (Fig. 2). For example, veratryl alcohol is a secondary metabolite, synthesized de-novo by several fungi (but not *P. ostreatus*)^27^, and shown to regulate the ligninolytic system by inducing the transcription of laccases^28,29^ and manganese peroxidases^29^.

Based on our findings, we suggest that SSPs and the generated OE*ssp1* mutant, which enhance the production of AAO, AAD and VP enzymes families, which can be potentially beneficial for various biotechnological applications. As the generated strain has increased tolerance towards HMF, the fungus can be used directly to degrade the toxic chemicals produced during biofuel pretreatment processes. It may also improve the rate of lignin degradation, which is necessary for biological pretreatment of biofuel lignocellulosic raw materials, or production of biomass utilized as ruminant feed^30–32^. To the best of our knowledge this is the first report suggesting regulators of the lignin degradation system, and a novel role of SSPs in saprophytic fungi.

## Methods

### Fungal growth and experimental conditions

*P. ostreatus* monokaryon strain PC9 (Spanish Type Culture Collection accession number CECT20311), which is a protoclone derived by de-dikaryotization of the commercial dikaryon strain N001 (Spanish Type Culture Collection accession number CECT20600)^33^, was used throughout this study. Strains KD*ssp1* and OE*ssp1*, generated during this study (see below) were constructed in a PC9 background.

Fungal strains were grown and maintained in GP medium (2% glucose, 0.5% peptone (Difco, Franklin Lakes, NJ, USA), 0.2% yeast extract (Difco, Franklin Lakes, NJ, USA), 0.1% K_2_HPO_4_, and 0.05% MgSO_4_ · 7H_2_O). When required, 1.5% agar was added. The gene and protein expression as well as activity assays were conducted in samples of fungal biomass or cell free extracellular extracts prepared from cultures that were maintained in stationary 100 ml Erlenmeyer flasks containing 10 ml of liquid media. The inoculum used for all experiments was one disk (5 mm diameter) of mycelium obtained from the edge of a young colony grown on solid medium and positioned at the center of the Petri dish or a flask. Cultures were incubated at 28 °C in the dark.

### Gene expression analyses

Total RNA was extracted from culture biomass, first ground under liquid nitrogen with mortar and pestle, then homogenized with QIA shredder spin columns (Qiagen, Hilden, Germany) and purified from the lysate using the RNeasy Plus Mini Kit (Qiagen, Hilden, Germany). cDNA was synthesized using the High Capacity cDNA Reverse Transcription Kit (Applied Biosystems, Carlsbad, CA, USA). Gene expression analyses were performed on an ABI StepOne Real-Time PCR Sequence Detection System and software (Applied Biosystems, Foster City, CA, USA), using Power SYBR Green PCR Master Mix (Applied Biosystems, Foster City, CA, USA). The PCR volume was 10 μl, using 20 ng of total cDNA and 300 nM oligonucleotide primers (Table S4). The thermal cycling conditions were as follows: an initial step at 95 °C for 20 s and 40 cycles at 95 °C for 5 s, 60 °C for 30 s, followed by a denaturation step to verify the absence of unspecific products or primer dimmers. To monitor the expression of *ssp*s 50 nM of primers were used and the thermal cycling was 40 cycles at 95 °C for 5 s, 64 °C for 60 s. The *β-tubulin* (ID: 117235) gene was used as the endogenous control. The primer efficiency levels of the genes were with the range of 90% to 110%. Amplification data were compared on the basis of the of ΔCT method and presented as 2^−ΔCT^ or ΔΔCT method and presented as 2^−ΔΔCT^. Data was normalized with respect to *β-tubulin* and calculated where ΔCT = CT *target gene* − CT *β-tubulin*, and then ΔΔCT = ΔCT *treatment* − ΔCT *control*.

### Protein expression profiles

For extracellular protein analyses, culture fluids were filtered through Whatman No. 1 filter paper followed by 0.45-μm mixed cellulose ester filter paper (Whatman, Buckinghamshire, UK). The sample was then concentrated using a 10-kDa cutoff PM-10 membrane (Millipore, Amicon Division, Billerica, MA, USA) and treated with cOmplete (Roche Applied Science, Mannheim, Germany), after concentration. For intracellular protein extraction, mycelial samples were frozen in liquid nitrogen, pulverized, and suspended in lysis buffer (1 M sorbitol, 10 mM HEPES (pH 7.5), 5 mM EDTA, 5 mM EGTA, 5 mM NaF, 0.1 M KCl, 0.2% Triton X-100, cOmplete (Roche Applied Science, Mannheim, Germany) and homogenized. The homogenates were centrifuged for 40 min at 10,000 × *g* at 4 °C, and the supernatants were recovered and stored at −70 °C until analyzed.

Protein concentration was determined using the BioRad protein assay kit (BioRad, Hercules, CA, USA). Proteins were separated on a NuPAGE 4% to 12% Bis-Tris gel in MES-SDS running buffer (Invitrogen, Grand Island, NY, USA) and visualized with Coomassie R-250 (0.125%). The sample was subsequently analyzed by HPLC/mass spectrometry/mass spectrometry (LC-MS/MS) in an Orbitrap (Thermo Scientific, Waltham, MA, USA) mass spectrometer and identified by Sequest 3.31 software against the JGI genome database of *P. ostreatus* PC9 v1.0 (http://genome.jgi-psf.org/PleosPC9_1/PleosPC9_1.home.html) at The Smoler Proteomics Center of The Israel Institute of Technology (Technion).

### Enzymatic activity assays

Aryl Alcohol oxidase (AAO) activity was assayed spectrophotometrically, as the oxidation of veratryl alcohol (3,4-dimethoxybenzyl) to veratraldehyde, monitored at 310 nm (*ε*_310_ = 9,300 M^−1^ cm^−1^). The reaction mixtures contained 1 mM veratryl alcohol in 50 mM potassium phosphate, pH = 6. The assay was conducted in a volume of 200 μl in microtiter plates at 30 °C, using the Synergy HTX Multi-Mode Microplate Reader (BioTek, Winooski, USA). An enzyme unit was defined as the amount enzyme producing 1 μmol of product per minute.

### Construction of KD*ssp1*

The construct for RNAi silencing of *ssp1* was designed with inverted 160-bp repeats, corresponding to 112-271 bp of *ssp1* coding sequence, separated by a 213 bp loop (linker region), under the control of *sdi1* expression signals^13^. The construct was inserted into pCS22 (Genscript, Nanjing, China) using the *Nhe*I and *Age*I restriction sites. The restriction sites were used to facilitate the introduction of the RNAi construct into pTMS1^13^, harboring the carboxin resistance gene, *Cbx*^R^, under the control of *sdi1* expression signals; the resulting plasmid was designated pDF3.

### Construction of OE*ssp1*

The *ssp1* sequence was found in the JGI genome database of PC9 v1.0 (http://genome.jgi-psf.org/PleosPC9_1/PleosPC9_1.home.html) as protein ID 65712. A restriction-free cloning technique^34,35^ was used to replace the *vp1* (previously referred as *mnp4*) with *ssp1* in pTMS12^36^. The *ssp1* gene was amplified from genomic DNA, using primers pTMS12-65712F and 65712-pTMS12R designed with www.rf-cloning.org^37^. The reaction was performed with Phusion™ High-Fidelity PCR Master Mix (Thermo Scientific, USA). Generating *ssp1* with promoter and terminator regions from *β-tubulin* with conjunct *ssp1* expressions and carboxin-resistance (*Cbx*^R^) cassette designated pDF6.

### Fungal Transformation

Transformation was performed based on the PEG-CaCl_2_ protocol previously adapted for *P. ostreatus*^13,36^. Carboxin was used as a selection marker, and resistance was conferred via introduction of the carboxin-resistance cassette (*Cbx*^R^) in either pDF3 or pDF6. Competent protoplasts were produced by digestion of vegetative mycelium of *P. ostreatus* from YMG liquid culture with lytic enzymes. The lytic enzyme solution consisted of 2% (w/v) Lysing enzymes from *Trichoderma harzianum* (Sigma-Aldrich) and 0.05% (w/v) Chitinase from *T. viride* (Sigma-Aldrich) in 0.5 M sucrose as an osmotic stabilizer. The protoplasts were washed (by centrifugation at 450 *g*, 8 min, 4 °C) in STC solution (18.2% w/v sorbitol, 50 mM Tris-HCl pH 8.0, 50 mM CaCl_2_, 0.5 M sucrose). Then, 2 ml protoplasts were mixed with 100 µl transforming DNA (300 ng/µl), 150 µl heparin solution (Sigma-Aldrich) (5 mg dissolved in 1 ml STC solution), and 300 µl single-strand λ phage carrier DNA (Fermentas) (500 µg/ml, after denaturation at 95 °C for 5 min and immediate transfer to ice). After 40 min of incubation on ice, 10 ml PTC solution (40% w/v PEG#4000, 50 mM Tris-HCl pH 8.0, 50 mM CaCl_2_, 0.5 M sucrose) was added, and the mixture was incubated for 20 min at room temperature. The mixture was then plated on selective solid YMG regeneration medium, containing 0.5 M sucrose and carboxin at a final concentration of 2 mg/l. Transformants were isolated after 10-16 days of incubation at 28 °C. Transformant stability was verified by three successive transfers to solid medium with carboxin selection drug (2 mg/ml), after which the transformants were transferred to solid culture medium lacking the antibiotic.

### cDNA libraries preparation and RNAseq

The mRNA from biological triplicates was isolated from 10 µg total RNA samples using Dynabeads Oligo(dT)25 (Invitrogen) and fragmented by the RNA Fragmentation Reagents kit (Ambion). The mRNA fragments were precipitated with 0.1 vol of 3 M sodium acetate and 2 vol of cold 100% ethanol, and washed with 70% ethanol. The precipitate was resuspended in Tris-EDTA buffer. The first cDNA strand was prepared using the SuperScript III First Strand Synthesis kit (Invitrogen) and [d(N6)] random primers (Invitrogen), and the second cDNA strand was prepared using the Second strand buffer (Invitrogen), according to the manufacturer’s instructions.

For the RNAseq experiments, the double-stranded cDNAs were end-labeled with different adaptors using the TruSeq DNA LT sample prep kit (Illumina), according to the manufacturer’s instructions. The end-labeled cDNAs of about 200 bp were purified by electrophoresis on a 2.5% low-melting point agarose gel (Qiagen, CA). The gel-purified cDNA libraries were amplified by PCR (TruSeq v2 LT sample prep kit PCR, Illumina), quantified, and checked for quality on an Agilent 2100 Bioanalyzer, and sequenced as single-end 50 bp reads on an Illumina Genome Analyzer GX platform.

### RNAseq data analysis

The sequence was mapped to the ORFs of the genome of *P. ostreatus* v1.0 (with Tophat v2.04 software^38^ and normalized expression determined for each gene as fragments per kilobase or exon model per million mapped fragments (FPKM) with the Cufflinks program v2.02^39^. The statistical significance differences in FPKM were determined by the cuffdiff module of the cufflinks program with only log2 > 1 fold change in expression with adjusted P-values below 0.01 after false determination rate correction were considered significant. The protein coding sequences of PC9 v1.0 were annotated by a protein blastp search to the non-redundant protein database at the NCBI using Blast2GO v2.8^40^ and e-value cutoff of 1e-5. Gene Ontology (GO) terms were used to assign biological annotation to each gene. Enrichment of GO terms for biological processes was by a Fisher’s exact test with an adjusted P-value < 0.05 as significant after a multiple testing correction.

## Electronic supplementary material

Supplementary Information

Table S1

Table S2

Table S3

Table S4

Table S5

Table S6
