# Author Correction: A role for small secreted proteins (SSPs) in a saprophytic fungal lifestyle: Ligninolytic enzyme regulation in *Pleurotus ostreatus*

**DOI:** 10.1038/s41598-018-21904-x

**Published:** 2018-03-06

**Authors:** Daria Feldman, David J. Kowbel, N. Louise Glass, Oded Yarden, Yitzhak Hadar

**Affiliations:** 1 The R.H. Smith Faculty Agriculture, Food and Environment, The Hebrew University of Jerusalem, Department of Plant Pathology and Microbiology, Rehovot, 76100 Israel; 2 University of California at Berkeley UC Berkeley, Department of Plant and Microbial Biology, 111 Koshland Hall, Berkeley, California 94720 USA

Correction to: *Scientific Reports* 10.1038/s41598-017-15112-2, published online 06 November 2017

In the original version of this Article, Supplementary Tables S1–S6 were published in an incorrect format. These errors have been corrected in the Supplementary Information that now accompanies the Article.

