# Supplementary material for: A role for small secreted proteins (SSPs) in a saprophytic fungal lifestyle: Ligninolytic enzyme regulation in Pleurotus ostreatus
Source: Sci Rep. 2017 Nov 6;7:14553. doi: 10.1038/s41598-017-15112-2 (PMC5674062; doi:10.1038/s41598-017-15112-2)

A role for small secreted proteins (SSPs) in a saprophytic fungal lifestyle: Ligninolytic enzyme regulation in *Pleurotus ostreatus*

Daria Feldman[1], David J. Kowbel[2], N. Louise Glass[2], Oded Yarden[1] and Yitzhak Hadar[1]*

[1]The R.H. Smith Faculty Agriculture, Food and Environment, The Hebrew University of Jerusalem, Department of Plant Pathology and Microbiology, Rehovot, 76100, Israel;

[2]University of California at Berkeley UC Berkeley, Department of Plant and Microbial Biology, 111 Koshland Hall, Berkeley, California 94720, USA

*Address correspondence to Yitzhak Hadar, yitzhak.hadar@mail.huji.ac.il

Supplementary information

Whole genome transcriptomic analysis of *Pleurotus ostreatus* exposed to HMF

We preformed RNA seq on 6 days cultures of *P. ostreatus*, after an exposure of 24 hours to 30mM of HMF. The results showed 577 genes whose expression was significantly increased when *P. ostreatus* was exposed to HMF, and 182 genes whose expression was down regulated (Table S2). Annotation of protein coding genes in the *P.* ostreatus genome was performed using Blast2GO (41).

The biological processes that increased following exposure to HMF included polysaccharide metabolic processes, carbohydrate catabolic processes and part of a parent carbohydrate metabolic process (GO:0005975) that contain 30 genes. Energy derived by oxidation of organic compounds is part of the cellular oxidation-reduction process (GO:0055114) that has 55 gene sequences associated with it. Amino acid transmembrane transport, carboxylic acid transport, nitrogen compound transport and ion membrane transport are part of the parent transport process (GO:0006810). Transcriptional processes including regulation of transcription, DNA dependent (GO:0006355), gene expression (GO:0010467) were also up-regulated. Monocarboxylic acid metabolic process (GO:0032787), carboxylic acid catabolic process (GO:0046395), dicarboxylic process , alpha-amino acid metabolic process (GO:1901605) and alcohol metabolic process (GO:0006066) are all upregulated and are part of a parent small molecule metabolic process (GO:0044281) that has 27 gene sequences associated with it. The processes that were significantly up regulated by exposure of *P. ostreatus* to HMF include oxidation-reduction (adjusted p-value 1.8e-8), transmembrane transport (adjusted

p-value 5.0e-3) carbohydrate metabolic process (adjusted p-value 3.0e-2) and acetyl-CoA metabolism adjusted p-value 3.0e-2) GO terms for molecular functions oxidaoreductase activity (GO:0016491) and its parent term catalytic activity (GO:0003824) were also enriched. No terms for cellular components were enriched. Only one-carbon metabolic process (GO:0006730) was significantly down regulated by HMF (adjusted p-value 1.13e-3) and had 4 genes down regulated (117150, s-adenosyl-l-homocysteine hydrolase; 92148, methionine adenosyltransferase; 117719, glycine hydroxymethyltransferase and 98707, methylenetetrahydrofolate reductase). These are involved in highly reactive single carbon transfers during amino acid or nucleotide metabolism (Fig. S1, Fig. S2, Fig. S3, Table S5 and Table S6).

Figure S1: GO terms associated with biological processes that are up regulated upon exposure to 5-HMF in *P. ostreatus.*

GO terms were associated with all protein coding genes using the program Blast2Go v 2.8 (6) and a multilevel pie chart generated from genes that were up regulated with a log2 value > 1.0 and p-adjusted value < 0.01 and a node score > 3.0.0. Node values were determined for each GO term as a function of sequences associated with each child term and the distance to each node of the child term. Only terminal nodes are presented in the pie chart with the node score for each biological process.

Figure S2: Enrichment of GO terms by Fisher's exact test upon exposure to HMF.

A one tailed fisher's exact test was used and only GO terms that were <0.05 after muliple testing hypothesis were considered significant. The % sequences are a ratio (# test or reference sequences / sum of test or reference sequences and not-annnotated sequences) as a percentage. Red bars are biological processes, green bars are molecular functions, blue bars are reference sequences for each GO term.

Figure S3: Directed acyclic graph (DAG) of hierarchy of GO terms associated with biological processes containing genes up regulated by HMF.

The node score for each GO term is calculated as a function of all the sequences associated with the term and the number of edges at each node. The score was weighted by distance using an alpha (α) level of one. Octagons represent direct annotated GO terms and squares represent intermediate GO terms.

Figure S4: Original gel of Figure 1B

The bottom of the gel was cropped and presented in Fig. 1B

Figure S5: Original gel of Figure 5A

Lanes 1-3 were cropped for Figure 5A from S5. Other lanes belong to non-related experiment.

Figure S6: Original gel of Figure 5B

Lanes 1-3 were cropped for Figure 5B from S6. Other lanes belong to non-related experiment.

Table S1

Expression of ligninolytic genes, by RNA-seq in *P. ostreatus*, after exposure to 30 mM of HMF of a 6 days old culture. Diffrentially expressed genes are marked in red.

Table S2

Expression of genes by RNA-seq in *P. ostreatus*, after exposure to 30 mM of HMF of a 6 days old culture.

Table S3

Homologues of the *Pleurotus* SSP family are present in other fungi. The BLAST analysis was performed using [16,17]. Evalue< 1.0E-5.

Table S4

Oligonucleotides used in this study.

Table S5

Enrichment of GO terms upon exposure to HMF by Fisher's exact test.

Table S6

GO terms and the associated parent terms for biological processes that have genes up regulated upon exposure to HMF.

Fig S1

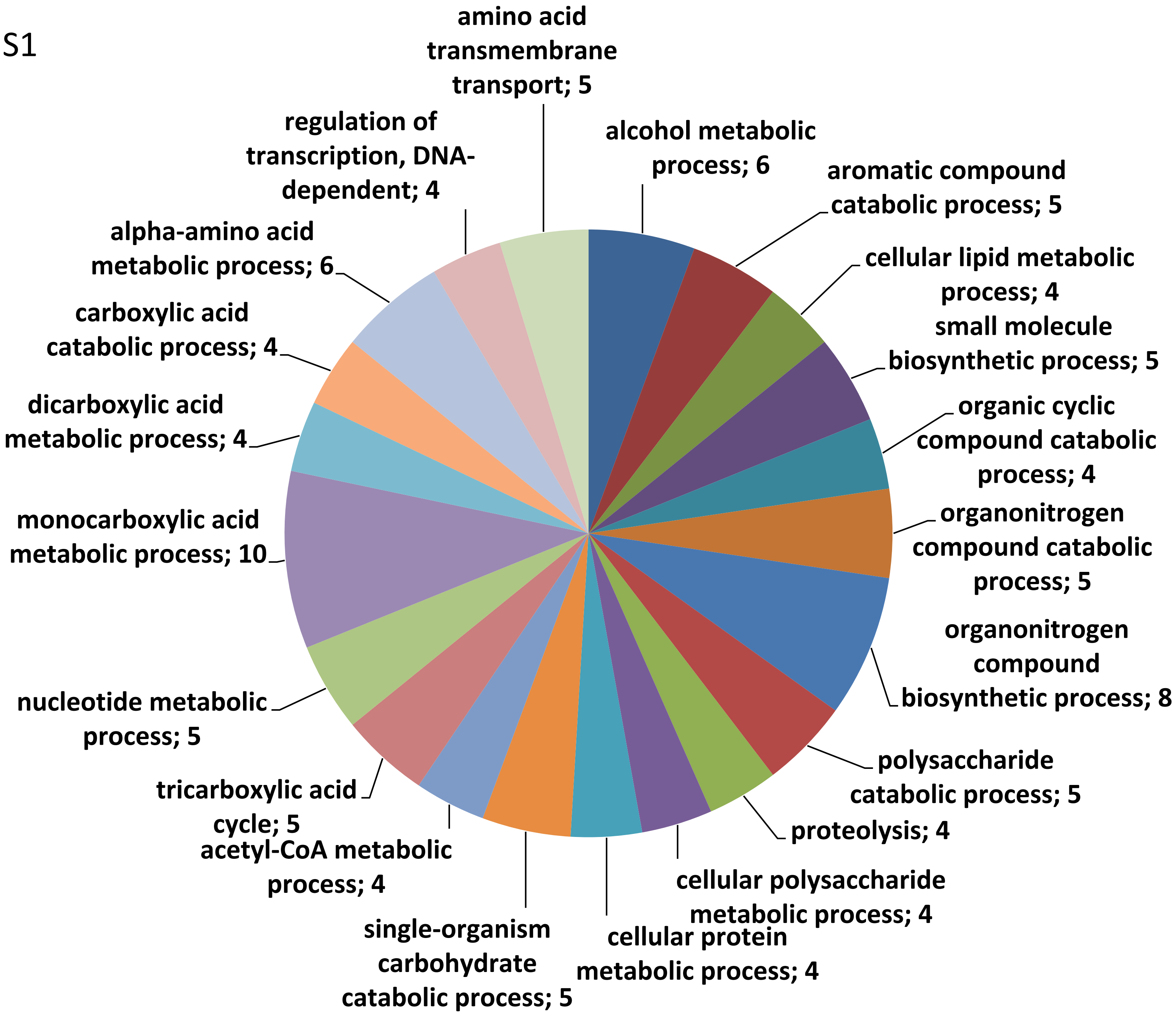
amino acid transmembrane transport; 5
alcohol metabolic process; 6
aromatic compound catabolic process; 5
cellular lipid metabolic process; 4
small molecule biosynthetic process; 5
organic cyclic compound catabolic process; 4
organonitrogen compound catabolic process; 5
organonitrogen compound biosynthetic process; 8
polysaccharide catabolic process; 5
proteolysis; 4
cellular polysaccharide metabolic process; 4
cellular protein metabolic process; 4
single-organism carbohydrate catabolic process; 5
acetyl-CoA metabolic process; 4
tricarboxylic acid cycle; 5
nucleotide metabolic process; 5
monocarboxylic acid metabolic process; 10
dicarboxylic acid metabolic process; 4
carboxylic acid catabolic process; 4
alpha-amino acid metabolic process; 6
regulation of transcription, DNA-dependent; 4

Fig S2

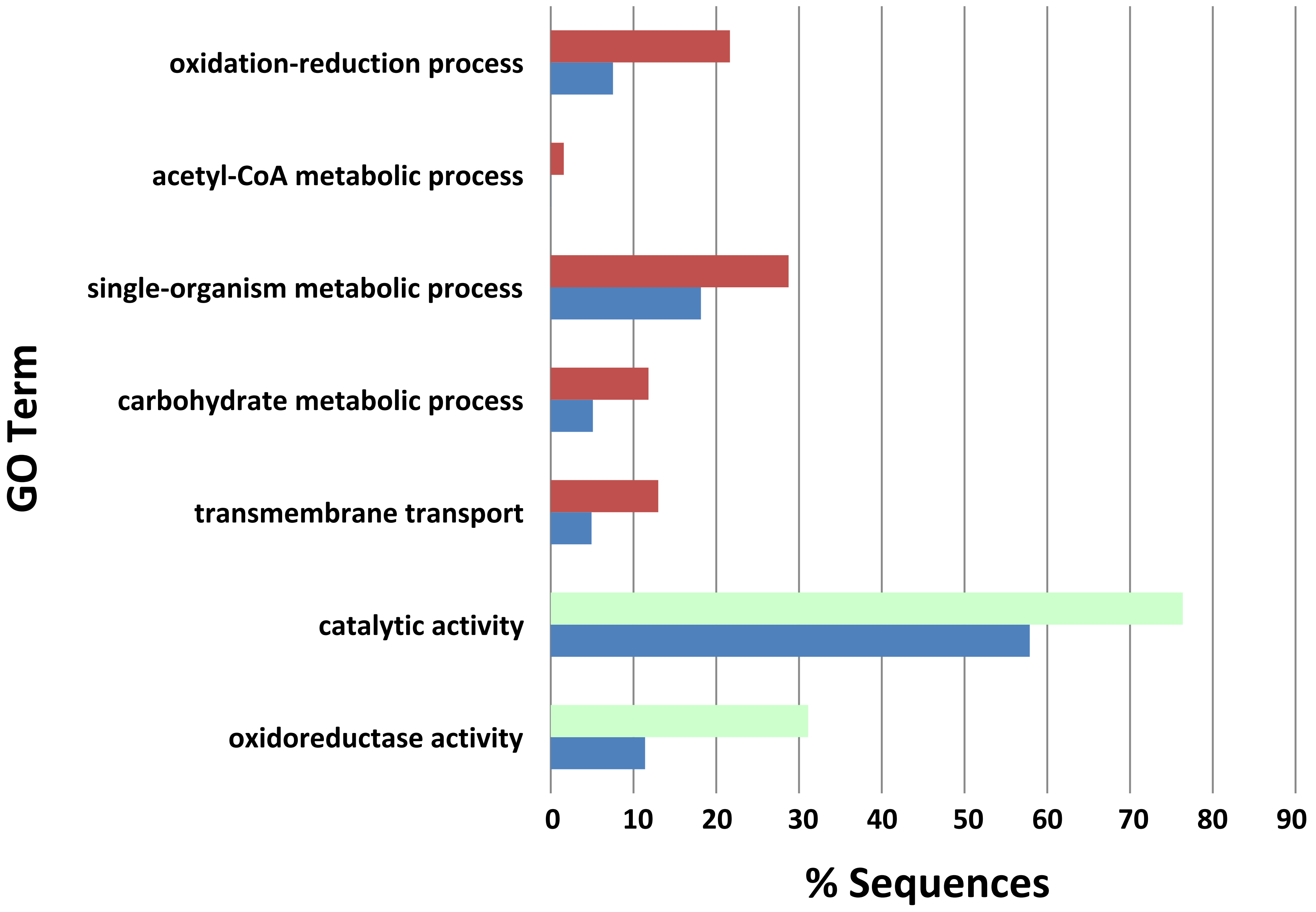

GO Term
oxidation-reduction process
acetyl-CoA metabolic process
single-organism metabolic process
carbohydrate metabolic process
transmembrane transport
catalytic activity
oxidoreductase activity
0
10
20
30
40
50
60
70
80
90
% Sequences

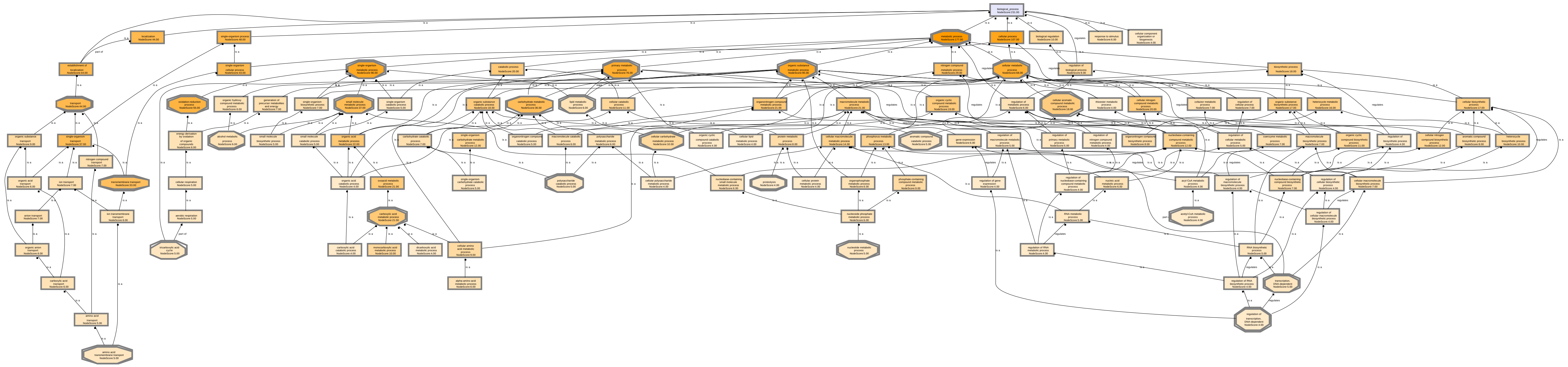

Combined Graph

Fig. S4

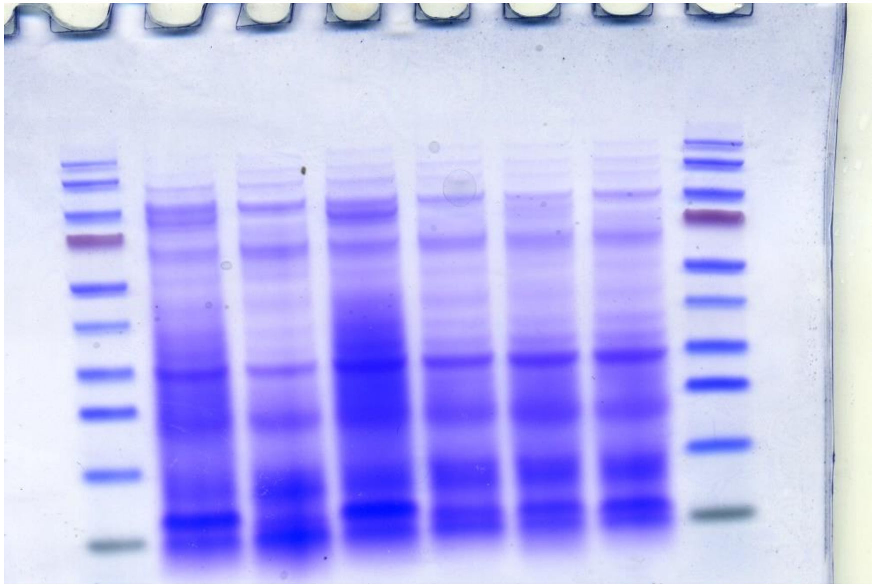

Fig. S5

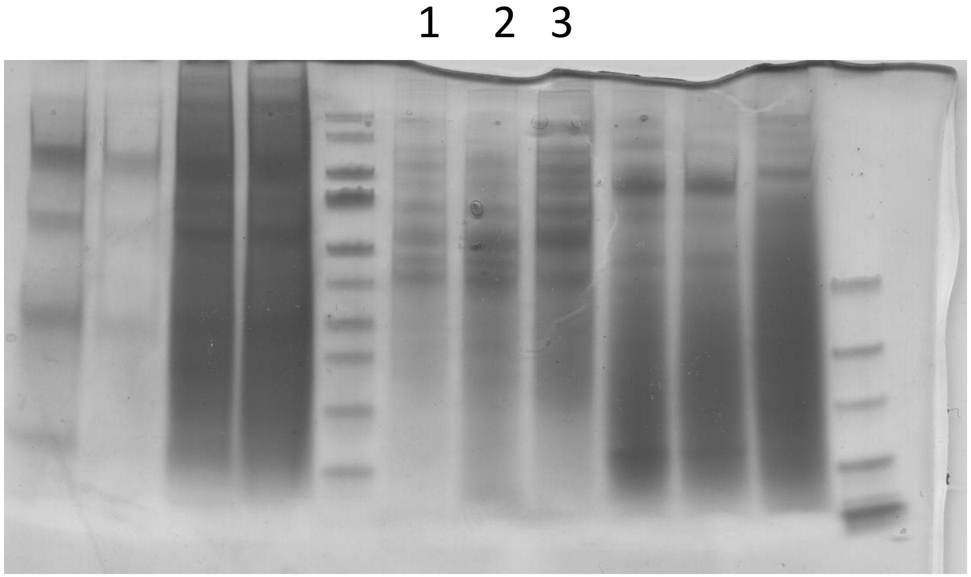

Fig. S6

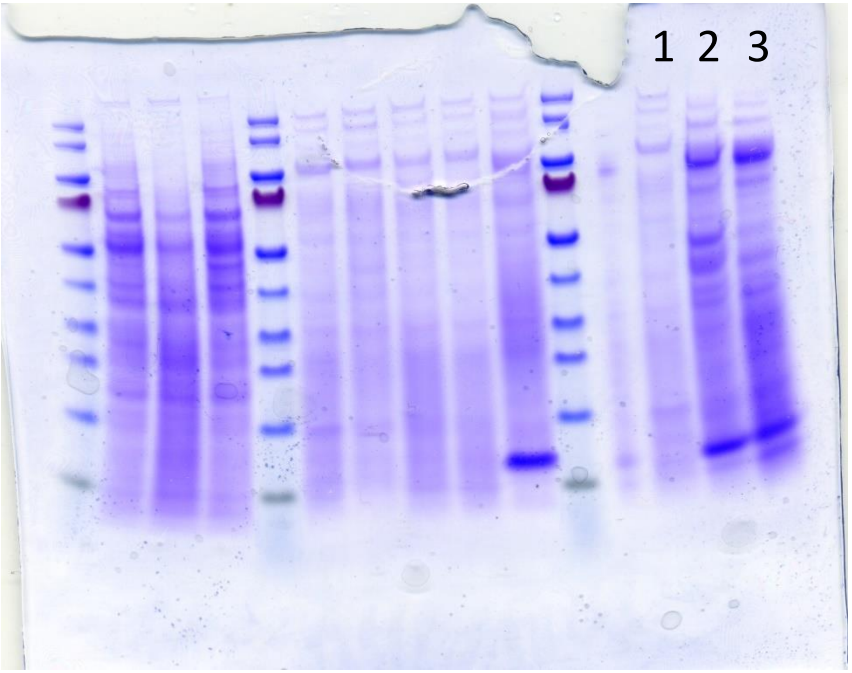

Supplement: Supplementary file 1 — Supplementary Information [file 41598_2017_15112_MOESM1_ESM.pdf]
