# Supplementary material for: A role for small secreted proteins (SSPs) in a saprophytic fungal lifestyle: Ligninolytic enzyme regulation in Pleurotus ostreatus
Source: Sci Rep. 2017 Nov 6;7:14553. doi: 10.1038/s41598-017-15112-2 (PMC5674062; doi:10.1038/s41598-017-15112-2)
Supplement: Supplementary file 2 — Table S1 [file 41598_2017_15112_MOESM2_ESM.pdf]

Aryl alcohol oxidases

Gene name	Protein_ID	Annotation	MeanFPKM_Con	MeanFPKM FUR	log2(foldchange)	p_value	q_value	significant	RQ
	129097	Glucose-methanol-cholin	1.2008	106.787	6.47459	0	0	yes	88.92949
aao4	114510	Glucose-methanol-cholin	11.2857	358.962	4.99127	0	0	yes	31.80695
aao1	69649	Glucose-methanol-cholin	22.1283	614.424	4.79527	0	0	yes	27.76643
	92777	Glucose-methanol-cholin	1.20162	29.0269	4.59434	0	0	yes	24.15651
aao6	121882	Glucose-methanol-cholin	18.4901	227.016	3.61797	0	0	yes	12.27771
aao5	116309	Glucose-methanol-cholin	8.48947	60.3357	2.82927	0	0	yes	7.107144
aao3	93955	Glucose-methanol-cholin	10.7239	49.0977	2.19482	0	0	yes	4.578325
	46589	Glucose-methanol-cholin	5.92211	20.943	1.82229	1.29E-14	5.56E-13	yes	3.536421
	45007	Glucose-methanol-cholin	17.4242	41.0181	1.23517	6.43E-10	1.77E-08	yes	2.354091
	101023	Glucose-methanol-cholin	1.07811	2.38961	1.14827	0.00524132	0.035432	yes	2.216479
	89272	Glucose-methanol-cholin	6.92316	14.0573	1.02181	0.0000135	0.000192	yes	2.030465
	49042	Glucose-methanol-cholin	6.8218	12.662	0.892283	0.000305621	0.003179	yes	1.856111
	92582	Glucose-methanol-cholin	2.95944	5.39262	0.865661	0.00425261	0.030057	yes	1.822174
	89266	Glucose-methanol-cholin	6.36824	10.4076	0.70867	0.00516192	0.035072	yes	1.634297
	86458	Glucose-methanol-cholin	87.9254	128.574	0.54825	0.00798417	0.049426	yes	1.462311
	91123	Glucose-methanol-cholin	1.08225	2.04183	0.915831	0.0276712	0.127552	no	1.886655
	65857	Glucose-methanol-cholin	6.27547	8.91704	0.506842	0.050947	0.196238	no	1.420936
aao2	82653	Glucose-methanol-cholin	8.45516	10.8705	0.362518	0.146372	0.39262	no	1.285668
	130566	Glucose-methanol-cholin	1.10899	1.4211	0.357755	0.424196	0.705544	no	1.28143
	105347	Glucose-methanol-cholin	1.66761	2.09109	0.326469	0.392413	0.677407	no	1.253941
	99302	Glucose-methanol-cholin	16.1224	20.1106	0.318889	0.123362	0.353388	no	1.24737
	129600	Glucose-methanol-cholin	18.2073	21.9339	0.268641	0.185547	0.450904	no	1.204673
	127507	Glucose-methanol-cholin	10.2572	11.5266	0.168336	0.464038	0.735156	no	1.123762
	91216	Glucose-methanol-cholin	5.14275	5.52793	0.104199	0.72579	0.896707	no	1.074897
	44590	Glucose-methanol-cholin	1.38019	1.4152	0.0361413	0.932722	0.97812	no	1.025368
	82668	Glucose-methanol-cholin	6.6553	6.78399	0.027631	0.915391	0.971105	no	1.019337
	45030	Glucose-methanol-cholin	3.72091	3.77882	0.0222801	0.943569	0.981791	no	1.015563
	117616	Glucose-methanol-cholin	370.712	373.291	0.0100041	0.970429	0.991586	no	1.006958
	64934	Glucose-methanol-cholin	1.71611	1.71586	-0.000210308	0.999575	0.999775	no	0.999854
	59433	Glucose-methanol-cholin	2.91475	2.90238	-0.00613807	0.985556	0.99567	no	0.995754
	96669	Glucose-methanol-cholin	5.04034	4.96212	-0.0225666	0.944629	0.982003	no	0.98448

	125919	Glucose-methanol-cholin	22.065	21.6635	-0.0264966	0.895629	0.965557	no	0.981802
	84776	Glucose-methanol-cholin	12.4701	11.5727	-0.107736	0.659898	0.862804	no	0.928043
	113150	Glucose-methanol-cholin	5.571	5.03542	-0.145824	0.606558	0.834622	no	0.903863
	88910	Glucose-methanol-cholin	16.5228	14.3434	-0.204073	0.339279	0.629746	no	0.868096
	100509	Glucose-methanol-cholin	1.44717	1.24377	-0.218516	0.616301	0.840155	no	0.859449
	83882	Glucose-methanol-cholin	27.7093	23.2659	-0.252153	0.194553	0.464498	no	0.839642
	88908	Glucose-methanol-cholin	1.40573	1.16085	-0.276132	0.531487	0.786073	no	0.825802
	87925	Glucose-methanol-cholin	15.5833	11.5209	-0.43574	0.0478271	0.187594	no	0.739314

aryl-alcohol dehydrogenases

Gene nam	Protein_ID	Annotation	MeanFPKM	MeanFPKM FUR	log2(foldchange)	p_value	q_value	significant	RQ
aad1	75413	Aldo/ketoreductase	368.409	3435.23	3.22103	0.00E+00	0	yes	9.324523
	85165	Aldo/ketoreductase	214.573	845.062	1.97759	0.00E+00	0	yes	3.938346
	123721	Aldo/ketoreductase	322.541	996.42	1.62727	1.26E-12	4.66E-11	yes	3.089279
	50158	Aldo/ketoreductase	107.432	284.389	1.40444	1.39E-12	5.07E-11	yes	2.64715
	50158	Aldo/ketoreductase	107.432	284.389	1.40444	1.39E-12	5.07E-11	yes	2.64715
	43757	Aldo/ketoreductase	84.3265	208.671	1.30717	1.93E-11	6.27E-10	yes	2.474557
	66752	Aldo/ketoreductase	16.2804	17.9757	0.142911	0.543967	0.793949	no	1.104131
	45854	Aldo/ketoreductase	7.66661	5.60404	-0.452122	0.142712	0.387329	no	0.730967

manganese peroxidases

Gene nam	Protein_ID	Annotation	MeanFPKM_Con	Mean FPKM_HMF	log2(foldchange)	p value	q value	significant	RQ
mnp1	115087	manganese peroxidase	5.37871	10.2972	0.93692	0.001094	0.00970155	yes	1.914437
mnp8	51713	manganese peroxidase	14.9073	15.3025	0.0377548	0.881746	0.961173	no	1.026515
mnp5	123383	manganese peroxidase	4.2144	4.28638	0.0244323	0.946019	0.982729	no	1.017079
mnp2	60432	versatile peroxidase	1.81791	1.56552	-0.215638	0.644642	0.854416	no	0.861165
vp1	116738	manganese peroxidase	1239.68	916.294	-0.436086	0.134836	0.374629	no	0.739137
mnp7	121638	manganese-repressed p	20.8073	15.2885	-0.444641	0.064132	0.230676	no	0.734767
mnp9	61491	manganese peroxidase	1.03393	0.73512	-0.492084	0.48356	0.750321	no	0.710997
mnp3	51690	manganese peroxidase	5.64419	3.95161	-0.514327	0.146349	0.39262	no	0.700119
mnp6	52120	manganese peroxidase	1.1954	0.834163	-0.519093	0.427009	0.707914	no	0.69781

laccase

Gene nam	Protein_ID	Annotation	MeanFPKM_Con	Mean FPKM_HMF	log2(foldchange)	p value	q value	significant	RQ
PoLACC6	81104	Multicopperoxidasetype1	4.29133	1.63E+01	1.92693	2.11E-13	8.32E-12	yes	3.802452
PoLACC3	123288	Multicopperoxidasetype1	4.05338	8.79E+00	1.11595	8.19E-05	0.000996	yes	2.167377
	65894	Multicopperoxidasetype1	2.0187	3.79233	0.90966	0.012879	0.072202	no	1.878603
	90573	Multicopperoxidasetype1	0.609377	1.03986	0.77098	0.204474	0.794684	no	1.706429
	81107	Multicopperoxidasetype1	0.96612	1.25581	0.378344	0.457816	0.73028	no	1.299849
	90812	Multicopperoxidasetype1	9.3328	11.7488	0.332128	0.148448	0.395319	no	1.258869
	90578	Multicopperoxidasetype1	1.75614	1.88074	0.0988953	0.815504	0.997124	no	1.070953
	60400	Multicopperoxidasetype1	1.05503	1.11896	0.0848722	0.868593	0.956095	no	1.060594
	90834	Multicopperoxidasetype1	9.18923	9.6821	0.0753753	0.78942	0.992151	no	1.053635
	116143	Multicopperoxidasetype1	4.07773	4.23583	0.0548792	0.871371	0.999149	no	1.038772
	81117	Multicopperoxidasetype1	1.2461	0.916268	-0.443573	0.350689	0.638603	no	0.735311
