# Supplementary material for: A role for small secreted proteins (SSPs) in a saprophytic fungal lifestyle: Ligninolytic enzyme regulation in Pleurotus ostreatus
Source: Sci Rep. 2017 Nov 6;7:14553. doi: 10.1038/s41598-017-15112-2 (PMC5674062; doi:10.1038/s41598-017-15112-2)
Supplement: Supplementary file 5 — Table S4 [file 41598_2017_15112_MOESM5_ESM.pdf]

Gene	Trascript ID	Primer designation	Sequence (5′ --->3′)
β-tubulin	117235	tub_117235_F1374	ACCAGTTCCACCACCAAGAG
		tub_117235_R1506	TGTTGTGCGTAAGGAAGCTG
aad-1	75413	AAD_75413_F72	AGGGTATTGTCCCCCAACTG
		AAD_75413_R191	TTTGAAGCTGGCCTCTTTGT
aao-1	69649	AAO_69649_F1495	GATGCCTCGATCTTCCCATA
		AAO_69649_R1688	CTTAATGTCGTCGGCCATCT
aao-2	82653	AAO_82653_F108	GTTGTCGGAGAACCTGGGTA
		AAO_82653_R234	AGGCAGCCTGAGGTACTGAA
aao-3	93955	AAO_93955_F1013	ATCCCCTCCTAGCCTGTGTT
		AAO_93955_R1110	GTCCGGTATGGTTTGCATTC
aao-4	114510	AAO_114510_F375	GGGATCCTCGGACAACTACA
		AAO_114510_R489	CGAAATGGTGTCGATTTGTG
aao-5	116309	AAO_116309_F1040	TCTTCGTCAACAGCAACCAG
		AAO_116309_R1149	AGGCCAAGTGGTTAGCAATG
aao-6	121882	AAO_121882_F941	CTGGCATCGGTGATCCTACT
		AAO_121882_R1041	CACCGCAATGATATGGTCAG
β-tubulin	117235	tubF543	GTGCGTAAGGAAGCTGAGGG
		tubR777	TGTGGCATTGTACGGCTCAAC
ssp	65712	SSP_65712_F163	TTTGGCTTCTCCGTGCAGTT
		SSP_65712_R488	CGTAGCTGGCCAACTTGG
vp-1	116738	MnP4F1269	TTGTTGGCTAGAGACCCCCAGA
		MnP4R1609	CAAGTGGGCCGCTCCGAC
ssp-1	65712	pTMS12-65712	CCATTCCTGAGATAGAACGCGACTAA
		65712-pTMS12	TAAGAGCAGGGAAAGGCGTCTCTTA<br>GTAGGTCTCGTAGCTGGC

Amplicon (bp)		Reference	Additional info
80			
130			
87			
146		Feldman et al., 2015	
117			Primers tub_117235_F1374 and tub_117235_R1506 were used for internal control
115	Gene expression		
129			
101			
228		Salame 2009	
343		This study	Primers tubF543 and tubR777 were used for internal control
186		Salame 2009	
567	contruction of pDF6	This study	Underlined nt represent sequence from pTMS12
